# Rapid Genome-Wide Location-Specific Polymorphic SSR Marker Discovery in Black Pepper by GBS Approach

**DOI:** 10.3389/fpls.2022.846937

**Published:** 2022-05-27

**Authors:** Ankita Negi, Kalpana Singh, Sarika Jaiswal, Johnson George Kokkat, Ulavappa B. Angadi, Mir Asif Iquebal, P. Umadevi, Anil Rai, Dinesh Kumar

**Affiliations:** ^1^Centre for Agricultural Bioinformatics, Indian Council of Agricultural Research-Indian Agricultural Statistical Research Institute, PUSA, New Delhi, India; ^2^Indian Council of Agricultural Research-Indian Institute of Spices Research, Kozhikode, India; ^3^Department of Biotechnology, School of Interdisciplinary and Applied Sciences, Central University of Haryana, Mahendragarh, India

**Keywords:** black pepper, diversity, genome, markers, polymorphic, web-resource

## Abstract

Black pepper (*Piper nigrum*), the “King of Spices,” is an economically important spice in India and is known for its medicinal and cultural values. SSRs, the tandem repeats of small DNA sequences, are often polymorphic in nature with diverse applications. For population structure, QTL/gene discovery, MAS, and diversity analysis, it is imperative to have their location specificity. The existing PinigSSRdb catalogs ~70K putative SSR markers but these are anonymous (unknown chromosomal location), based on 916 scaffolds rather than 26 chromosomes. Under this study, we generated ddRAD sequence data of 29 black pepper genotypes from all over India, being low-cost and most efficient technique for the identification of polymorphic markers. The major limitation of ddRAD with compromised/non-uniform coverage has been successfully overcome by taking advantage of chromosome-wise data availability. The latest black pepper genome assembly was used to extract genome-wide SSRs. A total of 276,230 genomic SSRs were mined distributed over 26 chromosomes, with relative density of 362.88 SSRs/Mb and average distance of 2.76 Kb between two SSRs. This assembly was also used to find the polymorphic SSRs in the generated GBS data of 29 black pepper genotypes utilizing rapid and cost-effective method giving 3,176 polymorphic SSRs, out of which 2015 were found to be hypervariable. The developed web-genomic resource, BlackP2MSATdb (http://webtom.cabgrid.res.in/blackp2msatdb/), is the largest and first reported web resource for genomic and polymorphic SSRs of black pepper, which is useful to develop varietal signature, coreset, physical map, QTL/gene identification, and MAS in endeavor of black pepper production.

## Introduction

Black pepper, one of the oldest spices globally used, belongs to the family *Piperaceae* (scientific name: *Piper nigrum; 2n* =*52*). It is widely known as “King of Spices” and is economically important spice in India, hence also termed as “Black Gold.” It is valued for its characteristic pungency and flavor (which is due to the alkaloid, *piperine*) along with many health benefits. It is perennial woody vine that gained its world-wide economic and cultural potential due to which its production and trade widened globally. Black pepper is known to have its origin in the Western Ghats of Southern India due to the humid climate, but now, it is also grown in tropical and sub-tropical regions of countries like Ethiopia, Vietnam, Brazil, Indonesia, India, Tajikistan, Sri Lanka, China, Malaysia, and Mexico. It is cultivated on total area of 259 thousand hectares with production of 65 thousand tons in India (Spice Board of India, Ministry of Agriculture and Farmers Welfare, Govt. of India), while globally it is cultivated on 749 thousand hectares with production of 1,103 thousand tons (FAO, 2019).

Microsatellites or simple sequence repeats (SSRs) are tandem repeats of small DNA sequences, often polymorphic in nature which serve in genetic analysis, mapping, gene tagging, and marker-assisted selection of linked desired economically important traits. Several attempts have been made in previous studies in development and characterization of black pepper microsatellite markers (Raghavan et al., 2010; Joy et al., 2011; Gordo et al., 2012; Hu et al., 2015; Jagtap et al., 2016; Wu et al., 2016; Jose et al., 2017) using various data sets including RNA-seq data. The first whole-genome SSR mining in black pepper was performed by Kumari et al. (2019) and presented in the form of a database, PinigSSRdb. This very first black pepper SSR database contains ~70 thousand putative SSR markers, but all are anonymous loci (unknown chromosomal location) as they are based on 916 scaffolds rather than 26 chromosomes. For population structure and diversity analysis, it is imperative to have SSR loci with known physical location (two–three SSRs per chromosome) having polymorphism information content (PIC) value >0.6 (four−12 alleles). The number of SSR loci can be drastically minimized, if markers are selected uniformly by taking the advantage of their known physical location over chromosome. Such selected markers can be a better diversity calculus for genomic and population variability with respect to random anonymous SSR loci (You et al., 2005). These chromosomes and location-specific SSR loci can be further used to trace the inheritance of particular chromosomal regions in molecular breeding programs from the foundation genotypes (Pestsova and Röder, 2002). Hence, the role of SSR markers in black pepper is immense including the study of its diversity, development of the germplasm core set, removal of duplicates, development of varietal signature, and low-cost polymorphic markers.

The latest genome assembly of black pepper provided by Hu et al. (2019) can be used prudently to cater the above critical needs. We used this latest assembly of black pepper to extract whole-genome SSRs with improved characterization and SSR-based assessment of genetic diversity among 29 genotypes of black pepper in India utilizing a rapid and cost-effective method to extract polymorphic SSRs. This was supplemented with the development of Black Pepper Polymorphic Microsatellite Database: *BlackP2MSATdb*, which provides the public access to the largest and first database of genomic and polymorphic SSRs of black pepper to be utilized by scientific community in the studies involving molecular breeding along with DNA fingerprinting, varietal identification, construction of physical map, identification of QTL, map-based gene cloning, marker-assisted selection, and evolutionary studies in black pepper and its related species.

## Materials and Methods

### Genomic DNA Extraction and GBS Data Generation

A total of 29 black pepper genotypes (released varieties, important germplasm accessions, and wild relatives) were maintained at Indian Council of Agricultural Research-Indian Institute of Spices Research, Kozhikode, Kerala, India (11.2986° N, 75.8405° E) (Supplementary File 1). The leaf samples were collected for DNA isolation from these 29 genotypes. The ddRAD genotyping-by-sequencing (GBS) data for these 29 genotypes of black pepper were generated for this study. The leaf samples were kept in liquid nitrogen. Total genomic DNA was isolated using CTAB extraction method (Saghai-Maroof MA). Quality and quantity checks of DNA were done by Qubit Fluorometer (Thermo Fisher Scientific Inc.). For determining A260/280 ratio, 1 μl of sample was loaded in Nanodrop (Thermo Fisher Scientific Inc.). The quality of samples was checked in 0.8% agarose gel electrophoresis along with Hind III marker for the presence of intact bands. In gDNA (100 ng), barcoded adapters (~0.1 pM) were added and double-digested with EcoRI and MseI enzyme for 2 h at 37 and 65°C for 20 min. The barcoded and common adapters were ligated to the sticky ends of the digested DNA with T4 DNA Ligase enzyme at 16°C overnight. The ligated products were pooled together. The pool of ligated products was size-selected to 150–650 bp using ampure beads. The pool was PCR-amplified to generate the final library. The library pool was analyzed in Bioanalyzer 2100 (Agilent Technologies) using high-sensitivity (HS) DNA chip as per manufacturer's instructions. The library pool was sequenced on Illumina NextSeq platform. Finally, the 29 RAD-based genotyping-by-sequencing (GBS) data libraries with pair-end reads were separated from library pool and passed through quality check.

### Quality Check and *de novo* Assembly of GBS Data Libraries

All the GBS libraries of 29 genotypes were passed through quality check using FastQC (Wingett and Andrews, 2018) keeping the default parameters, *viz.*, Phred score ≥30 and GC distribution >40%. *De novo* assembly was performed for each library using SPAdes-3.13.0 (Prjibelski et al., 2020) with default options. The *de novo* assembly for 29 samples of generated GBS data was done to find the polymorphic SSRs using the black pepper genome as reference (Hu et al., 2019). Simply mapping was not done to avoid missing of many SSRs from genome that are not represented in RefSeq.

### Genome-Wide Mining of SSR Markers and Their Primer Designing

In order to mine location-specific, genome-wide SSRs, black pepper reference genome was downloaded from http://cotton.hzau.edu.cn/EN/download.php (Hu et al., 2019). The black pepper genome has a total of 45 scaffolds, out of which 26 were considered as pseudo-chromosomes (Pn1–Pn26), while the remaining 19 scaffolds have been termed as PnU throughout the manuscript.

The SSRs were mined from the black pepper reference genome (Hu et al., 2019) and from GBS libraries of 29 genotypes using the perl script of MISA (MIcroSAtellite identification) tool (Beier et al., 2017). The criteria utilized for the identification were as follows: mononucleotide repeats motif with at least 10 repeats, dinucleotide with six, tri-, tetra-, penta- and hexa-nucleotide with five repeats (Thiel et al., 2003). Compound microsatellites were defined as those with the interval between two repeats motifs <=100 nucleotides as previous reports (Zhao et al., 2017).

For primer designing, SSRs mined from MISA along with their locations were processed using p3_in.pl perl script of MISA. Backward and forward primers from flanking sequences of each identified SSR were also extracted using Primer3 executable (Untergasser et al., 2012). The parameters used for primer designing are as follows: 18–27 bp primer length, 57–63°C melting temperature, 30–70% GC content, and 100–300 bp product size. The output obtained from Primer3 was processed using p3_out.pl perl script to extract the final set of primer sequences.

### Extraction of Genomic Frequency and Distribution of SSRs

Genomic frequencies per Mb and Kb of SSRs throughout genome and on all individual chromosomes were calculated considering size of the genome and sizes of all individual chromosomes, respectively. Genomic distribution into various genomic regions as exon, intron + intergenic regions, promoter (−1 Kb−100 bp), and TTS—transcription termination sites (−100 bp−1 Kb), of all black pepper genomic SSRs was extracted using HOMER (Heinz et al., 2010) utilizing black pepper reference genome annotation file.

### Polymorphic SSRs and Their Genomic Distribution

SSR mining was performed for all the 29 black pepper genotypes' libraries using MISA (Beier et al., 2017) along with primer designing using Primer3. The SSRs extracted from each genotype were compared to see the polymorphism with respect to the genome-wide SSRs extracted from the reference black pepper whole genome. The monomorphic SSRs along with polymorphic SSRs were extracted considering similar 5′ and 3′ 20 nucleotide flanking sequences along with variable number of SSR motifs using Perl scripts. Genomic distribution of polymorphic SSRs was extracted using HOMER (Heinz et al., 2010) utilizing black pepper reference genome annotation file. Hypervariable polymorphic SSR markers (those having ≥20 nucleotides) (Temnykh et al., 2001) were also extracted.

### Validation of Extracted SSRs

All the wet-lab validated anonymous SSRs from published literature were collected to be validated in our identified list of SSRs using e-PCR technique. For this validation of identified genomic SSRs, wet-lab validated 116 black pepper SSR primer pairs were collected (Menezes et al., 2009; Raghavan et al., 2010; Joy et al., 2011; Jagtap et al., 2016; Wu et al., 2016; Kumari et al., 2019; Meilawati et al., 2020). A total of 3682 primers of 736 SSRs extracted from black pepper transcriptome were used (Hu et al., 2015). These primers were mapped with 200 bp up- and down-stream of each of genomic SSRs of black pepper to find exact matches, taking cue from the concept of PrimerBLAST (https://www.ncbi.nlm.nih.gov/tools/primer-blast/index.cgi). In addition, this validation will add the location to the anonymous SSRs in black pepper genome.

### Functional Annotation of Genes Associated With Polymorphic SSRs

First, genes associated with polymorphic SSRs were extracted by Perl script using annotation file of black pepper reference genome. Then, functional annotation of extracted genes was performed using BLAST2GO and KEGG pathway analyses (Kanehisa, 2000).

### Development of BlackP2MSATdb Database

**Figure 1:**
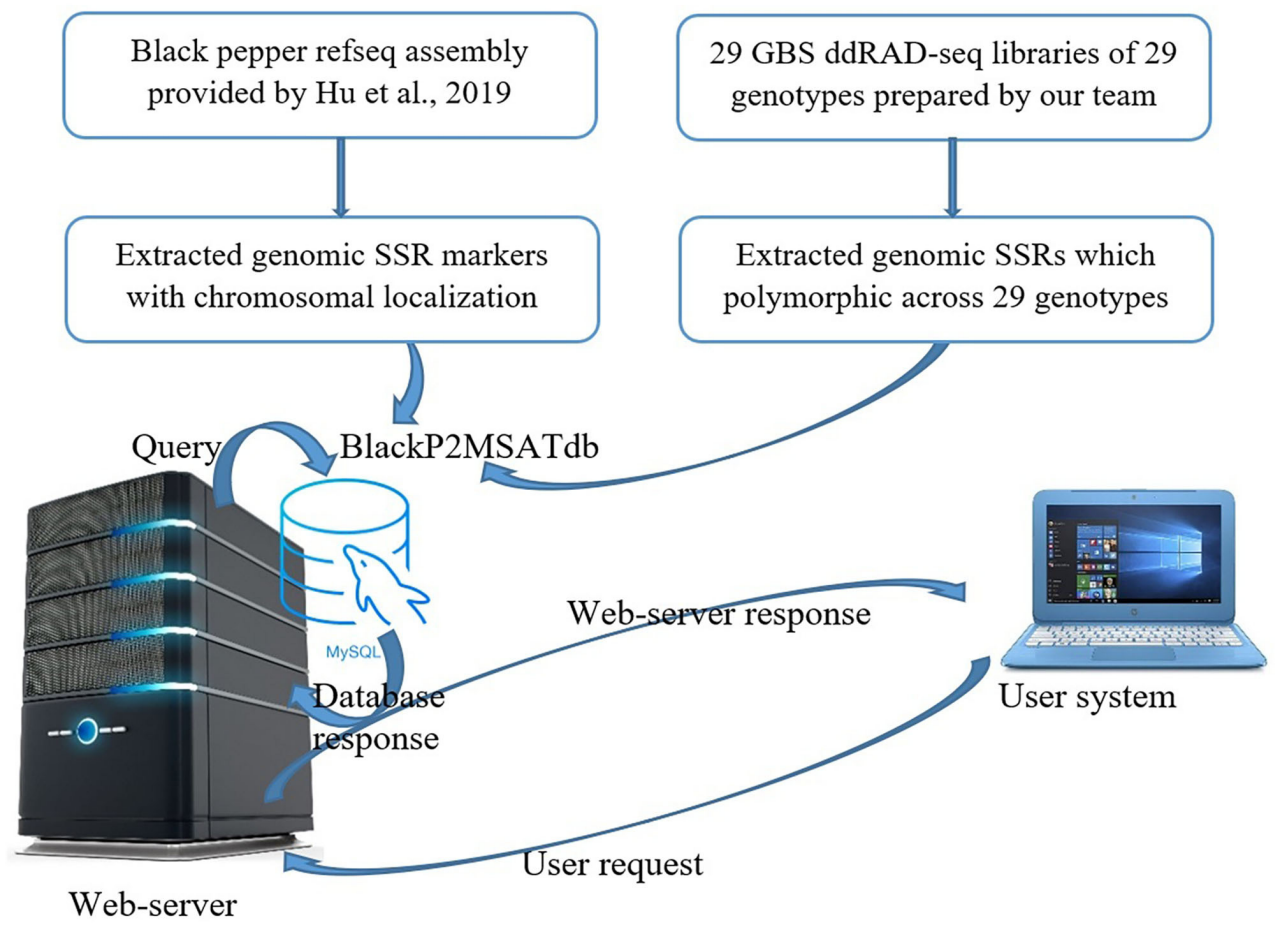
Schematic diagram of database development and data retrieval.

The Black Pepper Polymorphic Microsatellite database (BlackP2MSATdb) is a three-tier architecture-based relational database having client, middle, and database tier (Figure 1). The analysis results obtained in this study are cataloged in BlackP2MSATdb. A database was prepared in MySQL database, and its web interface was prepared in PHP and HTML, which includes the following steps of data retrieval: (a) A request generates from user's system to web server, (b) a query sends to MySQL database, (c) a database response generates and sends to web interface, and (d) finally, a web server response sends to user's system. Web hosting of this database was done by Apache2 server. BlackP2MSATdb includes details of SSR markers and polymorphic SSRs of black pepper obtained from GBS data analysis of 29 black pepper genotypes. The output of SSR markers gives the information of each marker in terms of its chromosome number, marker type, marker size, start position, end position, forward primer, reverse primer, and the link to genome.

## Results

### Quality Check and *de novo* Assembly of GBS Data Libraries

The pair-end reads of all the 29 GBS libraries were subjected to the quality check. The number of paired-end reads, GC %, assembled scaffolds, and the number of extracted SSR markers for each of the GBS libraries of 29 genotypes are shown in Table 1. The average number of reads was 1,842,097, while average GC %, scaffolds, and SSR markers were 39.74, 452,843, and 3,907, respectively. Genotype 4226 was found to have the largest number of reads (3,560,292) with 6,153 SSRs, while genotype KS had the largest number of mined SSRs (9,309). Genotype P2 was found to have least number of reads (213,631) and SSRs (1,007).

**Table 1:** Reads, quality, scaffolds, and extracted SSRs in each of GBS libraries of 29 black pepper genotypes.

**Genotype**	**PE reads**	**GC %**	**Scaffolds**	**SSRs**	**Genotype**	**PE reads**	**GC %**	**Scaffolds**	**SSRs**
2070	2,910,820	40	814,442	6,758	GIRI	2,818,577	40	716,463	5,910
4216	2,453,308	40	733,176	5,751	ME	539,793	39	240,190	2,227
4226	3,560,292	39	587,874	6,153	KS	1,776,541	39.5	924,676	9,309
5641	1,419,137	40.5	266,974	2,033	OP-P24	2,218,687	40.5	327,345	2,501
816	1,271,425	40	267,918	2,229	PAN	1,550,235	39	572,412	5,063
CE	293,718	40	136,099	1,118	PLD	2,498,978	39	361,046	3,098
CHERI	1,652,135	40	305,448	2,282	P.ATT	3,539,707	39.5	771,154	8,551
P1	2,219,162	39.5	316,786	2,408	POU	1,341,198	40	273,567	2,146
P2	213,631	41	82,713	1,007	SHAK	2,238,734	40	669,590	5,888
P3	861,024	39	378,171	3,384	SRE	2,293,560	40.5	393,255	2,956
P4	935,613	40	229,541	1,747	SUB	1,831,430	40	457,158	3,580
P5	1,162,094	39	290,715	2,447	VAD	2,174,345	40.5	621,325	4,788
P6	1,959,805	39	489,107	3,794	THE	3,059,600	39	685,171	5,902
P7	1,876,837	39.5	466,331	3,650	UTHI	1,616,470	40.5	319,043	2,491
P8	1,133,968	39	434,763	4,151					

### Genome-Wide Mining of SSR Markers and Their Primer Designing

**Figure 2:**
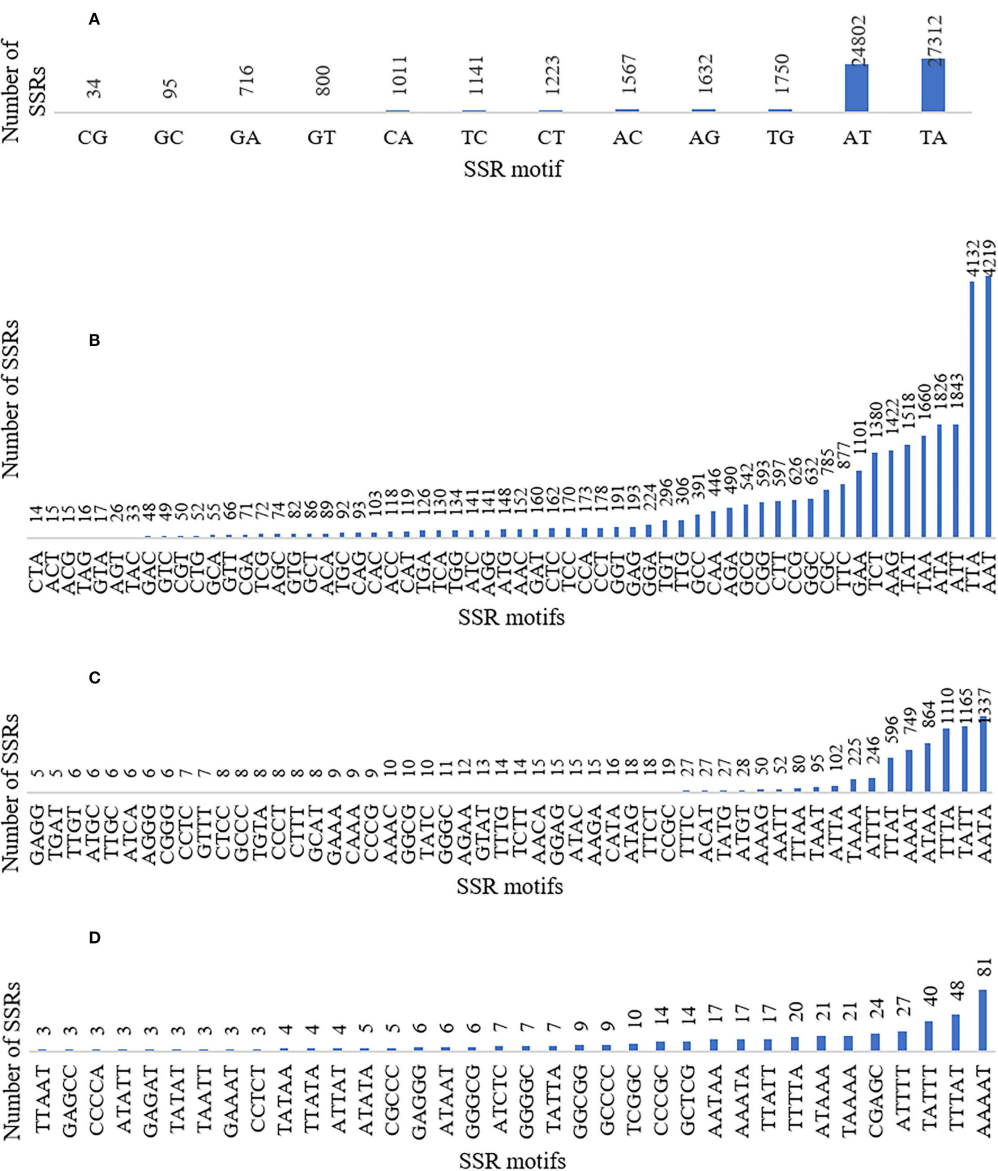
Graphical representation of **(A)** di- (included all motifs), **(B)** tri- (included all motifs), **(C)** tetra- (included motifs with ≥3 frequency), and **(D)** penta-nucleotide (included motifs with ≥5 frequency) SSR motifs with their frequencies and patterns.

The SSRs (mono- to hexa-nucleotide and compound SSRs) were mined from the black pepper reference genome (genome size ~761 Mb having 45 scaffolds) and GBS libraries of 29 genotypes using MISA tool. A total of 276,230 SSRs were identified, out of which 275,966 SSRs were found in 26 pseudo-chromosomes (termed as Pn1–26) and only 264 SSRs were found in the remaining 19 scaffolds. The genomic distribution of SSRs showed the abundance of SSRs in intergenic + intronic region (175,641), followed by promoter (70,396), TTS (28,861), and exonic regions (1,331). The average distance between two SSRs in black pepper genome was 2.76 Kb, and the frequency of SSRs per Mb was 362.88. A total of 457 types of mono- to hexa-nucleotide SSR motifs were among all detected SSRs. Out of these SSR motifs, 4, 12, 60, 136, 104, and 141 types belong to mono-, di-, tri-, tetra-, penta-, and hexa-nucleotide repeats, respectively (Table 2). Among 276,230 SSRs, the mononucleotide repeats were the most abundant in proportion and frequency per Mb (137,675, 49.84%) followed by the proportion and frequency of dinucleotide (62,083, 22.48%), compound (38,132, 13.80%), tri-nucleotide (29,560, 10.7%), tetra-nucleotide (7,297, 02.64%), penta-nucleotide (562, 0.20%), and hexa-nucleotide (920, 0.33%). Frequencies of a particular motif type (di-, tri-, tetra-, and penta-nucleotide) are represented in Supplementary Table S1. This table shows the frequency and pattern of di- (including all motifs), tri- (including motifs with ≥141 frequency), tetra- (including motifs with ≥10 frequency), penta- (including motifs with ≥3 frequency), and hexa-nucleotide (including motifs with ≥2 frequency) SSR motifs. A total of 12 and 60 SSR motifs extracted were di- and tri-types, respectively (Figures 2A,B), while 36 out of 136 tetra-nucleotide motifs were having ≥3 frequency (Figure 2C). A total of 52 out of 104 penta-nucleotide motifs had the frequency ≥ 5 (Figure 2D). Abundance of TA motif type (27,311) was observed, followed by AT (24,802), AAT (4,219), and TTA (4,132).

**Table 2:** Summary of SSRs in black pepper genome.

**Repeats**	**Number**	**Motif/pattern**	**Proportion %**	**Frequency (per Mb)**
Mono	137,676	4	49.84	180.83
Di	62,083	12	22.48	81.57
Tri	29,560	60	10.7	38.84
Tetra	7,297	136	2.64	9.59
Penta	562	104	0.20	0.74
Hexa	920	141	0.33	1.2
Compound	38,132	34,569	13.8	50.1

### Extraction of Genomic Frequency and Distribution of SSRs

A comprehensive overview of the mined SSRs was studied. Based on the overall size of black pepper genome, the overall frequency of occurrence of SSRs per Mb within the genome was found to be 362.88 SSRs. The average distance between two SSRs was 2.76 Kb.

**Figure 3:**
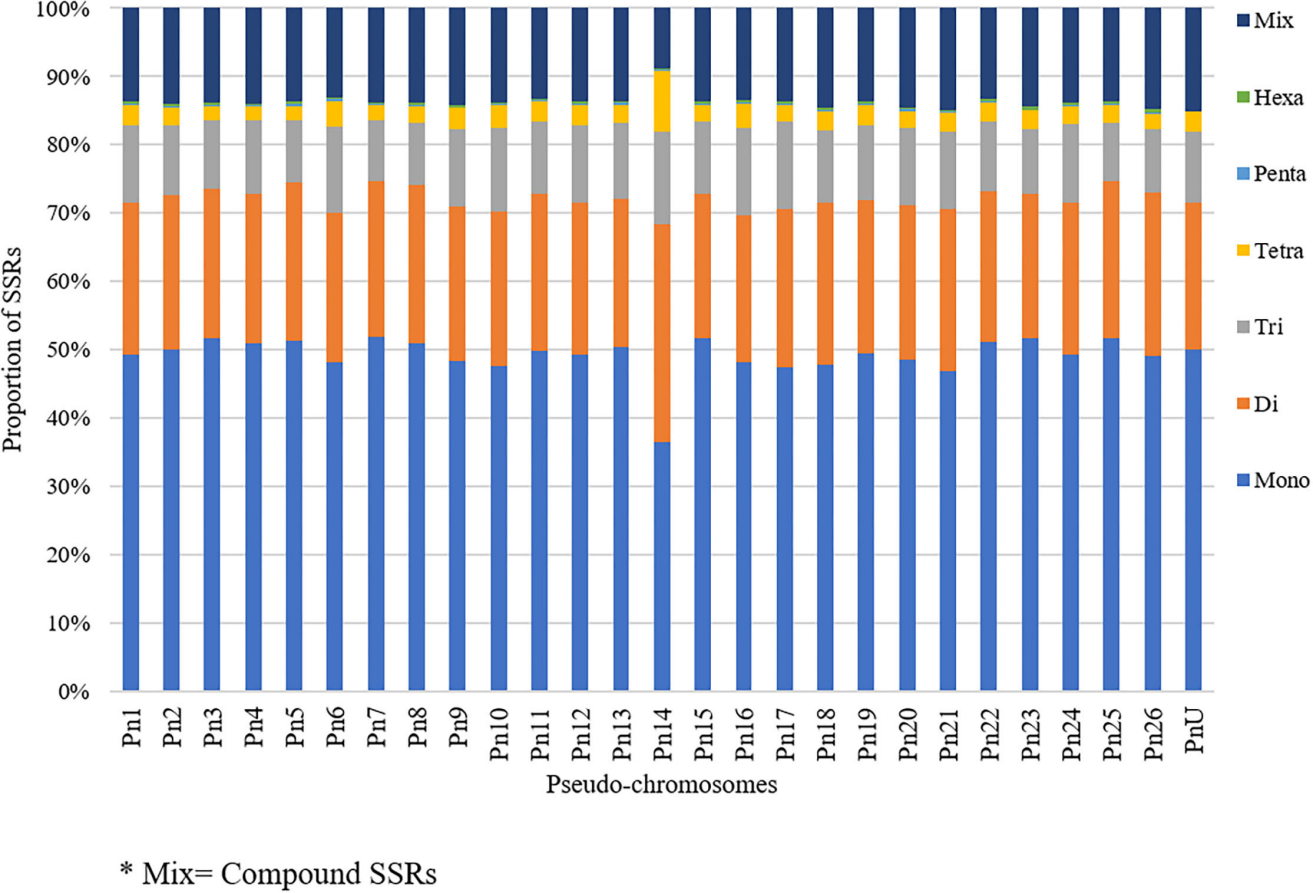
Chromosome-wise distribution of mono- to hexa-nucleotide SSRs and compound SSRs.

**Figure 4:**
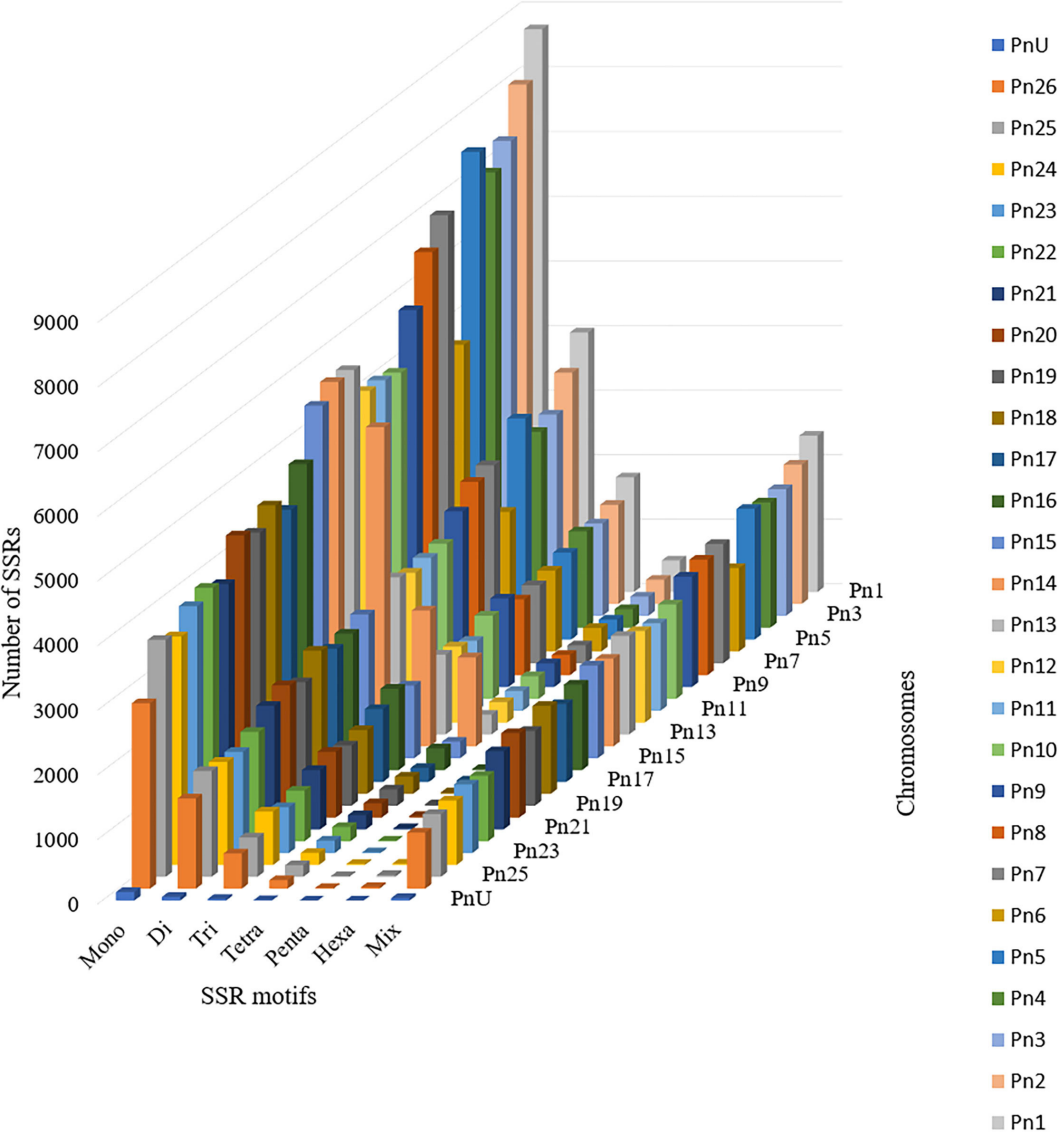
Distribution of SSR motif repeat number from mono- to hexa-nucleotide along with compound SSRs.

The stacked bar graph shows the proportions of SSRs for each chromosome with SSR motifs stacked as mono-, di-, tri-, tetra-, penta-, hexa-, and compound types (Figure 3), while Figure 4 delineates the chromosome-wise numbers of mono- to hexa-nucleotides SSRs in three dimensions. For each of the pseudo-chromosomes of black pepper, the frequency distribution of SSRs per Mb and the average distance between two SSRs in Kb were found (Table 3). It was seen that the pseudo-chromosome Pn1 had the largest number of SSRs (17501), followed by Pn2, Pn5, Pn3, Pn4, Pn7, Pn8, Pn9, Pn13, Pn10, Pn15, Pn12, Pn11, Pn14, and Pn6. The minimum number of SSRs was detected in PnU (280). The highest frequency of SSR markers was observed on Pn7 (398.4), while the least was on Pn6. The average distance between two SSRs is the maximum (3.73) in Pn6 and the least in Pn7.

**Table 3:** Distribution of SSRs along with frequency per Mb and distance between two SSRs in each of pseudo-chromosomes of black pepper.

Chromosome^*^	**SSRs**	**Frequency per Mb**	**Distance in Kb**	Chromosome^*^	**SSRs**	**Frequency per Mb**	**Distance in Kb**
Pn1	17,501	361.20	2.77	Pn15	10,558	367.65	2.72
Pn2	15,758	365.57	2.74	Pn16	9,814	350.88	2.85
Pn3	14,219	361.01	2.77	Pn17	8,873	344.83	2.9
Pn4	13,842	371.75	2.69	Pn18	9,335	364.96	2.74
Pn5	14,724	395.26	2.53	Pn19	8,529	343.64	2.91
Pn6	9,856	268.1	3.73	Pn20	9,003	370.37	2.7
Pn7	13,388	398.4	2.51	Pn21	8,089	363.64	2.75
Pn8	12,865	393.7	2.54	Pn22	7,671	346.02	2.89
Pn9	12,044	337.84	2.69	Pn23	7,401	362.32	2.76
Pn10	10,598	338.98	2.95	Pn24	7,180	361.01	2.77
Pn11	10,266	344.83	2.90	Pn25	7,102	389.11	2.57
Pn12	10,417	352.11	2.84	Pn26	5,850	392.16	2.55
Pn13	11,191	380.23	2.63	PnU	280	358.42	2.79
Pn14	10,105	348.43	2.87				

* *Chromosome = Pseudo-chromosome*.

### Polymorphic Markers, Their Genomic Distribution, and Hypervariable Polymorphic Markers

**Figure 5:**
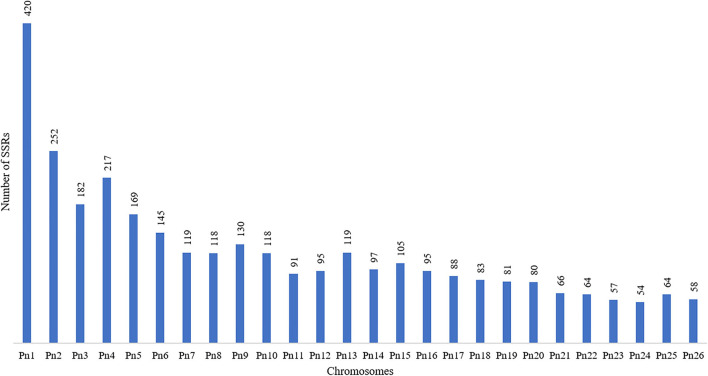
Distribution of polymorphic SSRs on different chromosomes of black pepper genome.

**Figure 6:**
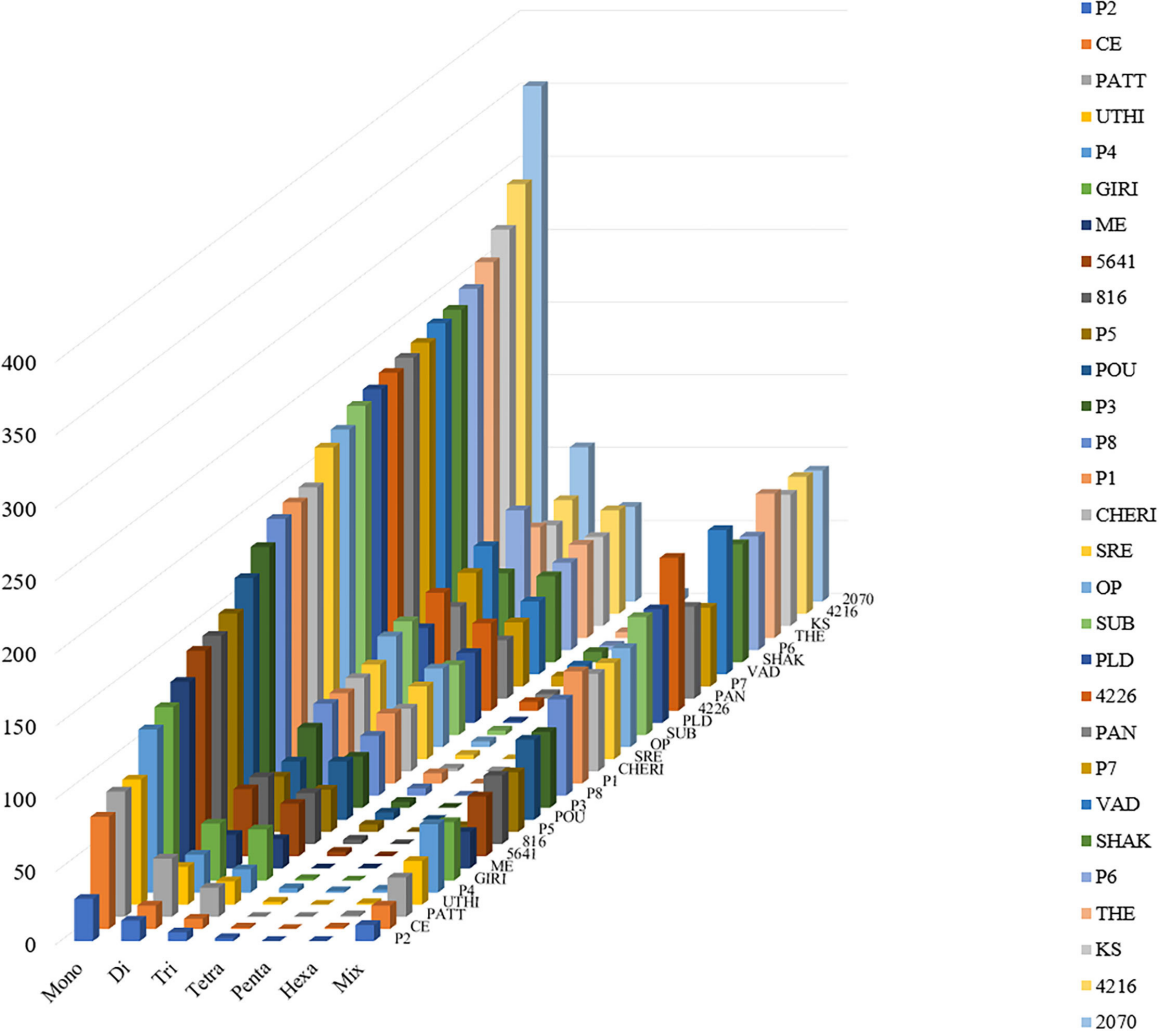
Distribution of polymorphic SSR motif repeat number from mono- to hexa-nucleotide along with compound SSRs (termed as Mix in figure). The y-axis shows the abundance of polymorphic SSRs that have different motif repeat numbers on x-axis in different genotypes (29 genotypes of black pepper) on z-axis which are discriminated by legends of different colors.

For the extraction of polymorphic SSRs, genomic SSRs extracted from black pepper reference genome were compared with the SSRs extracted from GBS libraries of 29 genotypes. A total of 5,949 genomic SSRs were found common in both black pepper reference genome and 29 genotypes collectively, out of which 2,773 genomic SSRs were monomorphic and 3,176 genomic SSRs were polymorphic. The number of monomorphic and polymorphic SSRs in each of the GBS libraries of 29 black pepper genotypes along with their genomic distributions is shown in Table 4. Figure 5 shows chromosome-wise distribution of polymorphic SSRs which obviates the abundance of polymorphic SSRs on chromosome Pn1, followed by Pn2, Pn4, and Pn3. Figure 6 shows the chromosome-wise distribution of mono-, di-, tri-, tetra-, penta-, hexa-nucleotide, and compound motifs of polymorphic SSRs found in each of the 29 genotypes. Out of all polymorphic SSRs, 2015 were hypervariable in reference genome and/or in the particular genotype where it is showing polymorphism. Table 5 shows mono-, di-, tri-, tetra-, penta-, hexa-nucleotide, and compound motif-wise distribution of hypervariable polymorphic SSRs. Chromosome-wise distribution of hypervariable polymorphic SSRs along with frequency per Mb and distance in Kb between two hypervariable SSRs is represented in Table 6.

**Table 4:** Monomorphic and polymorphic SSRs in each of the GBS libraries of 29 black pepper genotypes and their genomic distributions.

**Genotype**	**Mono^**1**^**	**Poly^**2**^**	**I+I^**3**^**	**E^**4**^**	**P^**5**^**	**TTS^**6**^**	**Genotype**	**Mono^**1**^**	**Poly^**2**^**	**I+I^**3**^**	**E^**4**^**	**P^**5**^**	**TTS^**6**^**
2070	630	619	379	0	171	69	GIRI	271	236	138	0	75	23
4216	596	541	356	0	118	67	ME	170	196	115	0	65	16
4226	590	482	291	2	135	54	KS	526	496	334	0	97	65
5641	314	269	164	0	70	35	OP-P24	407	424	261	1	106	56
816	274	274	162	0	79	33	PAN	428	405	267	0	98	40
CE	135	117	64	0	41	12	PLD	448	421	253	3	116	49
CHERI	412	374	245	0	92	37	P.ATT	76	173	89	0	67	17
P1	385	387	242	0	102	43	POU	318	303	188	0	80	35
P2	63	62	35	0	18	9	SHAK	506	453	274	0	120	59
P3	348	326	219	0	78	29	SRE	434	401	236	0	114	51
P4	273	207	121	0	61	25	SUB	458	433	251	0	47	135
P5	320	265	155	1	72	34	VAD	453	448	289	1	140	54
P6	506	487	310	0	130	47	THE	558	503	309	0	128	66
P7	439	419	254	0	123	42	UTHI	184	159	93	0	43	23
P8	390	365	224	0	95	46							

*1 = monomorphic SSRs; 2 = polymorphic SSRs; 3 = intergenic+intron; 4 = exon; 5 = promoter; 6 = transcription termination site*.

**Table 5:** Frequency of hypervariable SSRs with di-, tri-, tetra-, penta-, and hexa-nucleotide motifs along with frequency per Mb and distance in Kb between two hypervariable SSRs.

**Repeat motif**	**Frequency**	**Frequency per Mb**	**Distance in Kb**
Mono	126	0.17	6041.27
Di	849	1.12	896.58
Tri	547	0.72	1391.59
Tetra	57	0.07	108742.86
Penta	7	0.01	108742.86
Hexa	54	0.07	14096.30
Compound	375	0.49	2029.87

**Table 6:** Chromosome-wise frequency of hypervariable SSRs along with frequency per Mb and distance in Kb between two hypervariable SSRs.

**Chromosome**	**SSRs**	**Frequency per Mb**	**Distance in Kb**	**Chromosome**	**SSRs**	**Frequency per Mb**	**Distance in Kb**
Pn1	427	8.81	113.47	Pn14	54	1.86	537.64
Pn2	199	4.62	216.61	Pn15	64	2.23	448.33
Pn3	106	2.69	371.55	Pn16	46	1.65	607.57
Pn4	118	3.17	315.16	Pn17	35	1.36	734.88
Pn5	85	2.31	432.69	Pn18	61	2.39	418.94
Pn6	65	1.93	517.11	Pn19	35	1.41	708.82
Pn7	69	2.11	474.45	Pn20	59	2.43	411.60
Pn8	79	2.41	414.25	Pn21	48	2.16	463.76
Pn9	79	2.43	411.17	Pn22	38	1.71	583.36
Pn10	65	2.08	480.48	Pn23	28	1.37	730.09
Pn11	36	1.21	828.13	Pn24	33	1.66	601.61
Pn12	78	2.64	379.50	Pn25	24	1.32	759.35
Pn13	39	1.33	754.56	Pn26	45	3.02	331.26

### Validation of Extracted SSRs

For the validation of genomic-wide SSRs mined from black pepper reference genome, wet-lab validated and previously reported primers from eight studies were utilized as mentioned under methodology section. A total of 3,798 primer sequences (116 wet-lab validated SSRs from literature with one primer pair plus 736 SSRs with 5 primer pairs (forward and reverse) for most of SSRs from Hu et al., 2015) of 852 validated SSR repeats from various published literature were used for SSR validation. The validated microsatellites (both *in vivo* and *in silico*) used for *in silico* re-validation were taken from eight different studies represented by countries like China, Brazil, and India. Results obtained are shown in Table 7 along with SSR motifs and references of re-validated primers of 360 SSR repeats (primer IDs provided in Supplementary Table S2), out of which 330 SSRs were from Hu et al. (2015).

**Table 7:** Validated black pepper genome-wide SSR markers and their method of SSR discovery (The bold ones are the polymorphic SSRs, which could not be determined in case of SSRs listed in Kumari et al., 2019).

**References**	**Number of markers**	**Method of marker discovery**
Jose et al. (2017)	1 [(TCT)5]	PCR
Kumari et al. (2019)	19 [AT, TAT, TTA, (TA)T(TA), (AT)A(AT), (TC)(TA), GCG, AAT, AT, TTA, (TTC)(TCT), (TA)(TTTA), (TC)(AC), (TG)(TA), TA, TA, TTA, ATA, CCT]	PCR
Menezes et al. (2009)	5 [**(AC)19**, **(TG)14**, **(GT)13**, **(CA)13**, **(AC)5**]	PCR
Raghavan et al. (2010)	5 **[(AC)19**, **(TG)14**, **(GT)13, (CA)13**, **(AC)5**]	PCR
Jagtap et al. (2016)	6 [**(CT)4TT(CT)16**, **(AC)19, (TG)14**, **(GT)13, (CA)13**, **(AC)5**]	PCR
Wu et al. (2016)	3 [CTT (5), **CAT (7)**, **CTC (7)**]	PCR
Joy et al. (2011)	2[**(CT)4TT(CT)16**, **(AT)6(GT)24**]	PCR
Hu et al. (2015)	330 (47 polymorphic and 283 monomorphic)	*In Silico*

All the anonymous *in vitro* /wet-lab validated SSRs from published literature were validated *in silico* in our identified list of SSRs using e-PCR technique which is a kind of re-validation of wet-lab validated markers. In addition, this validation will add the location to those anonymous SSRs in black pepper genome.

### Functional Annotation of Genes Associated With Polymorphic SSRs

**Figure 7:**
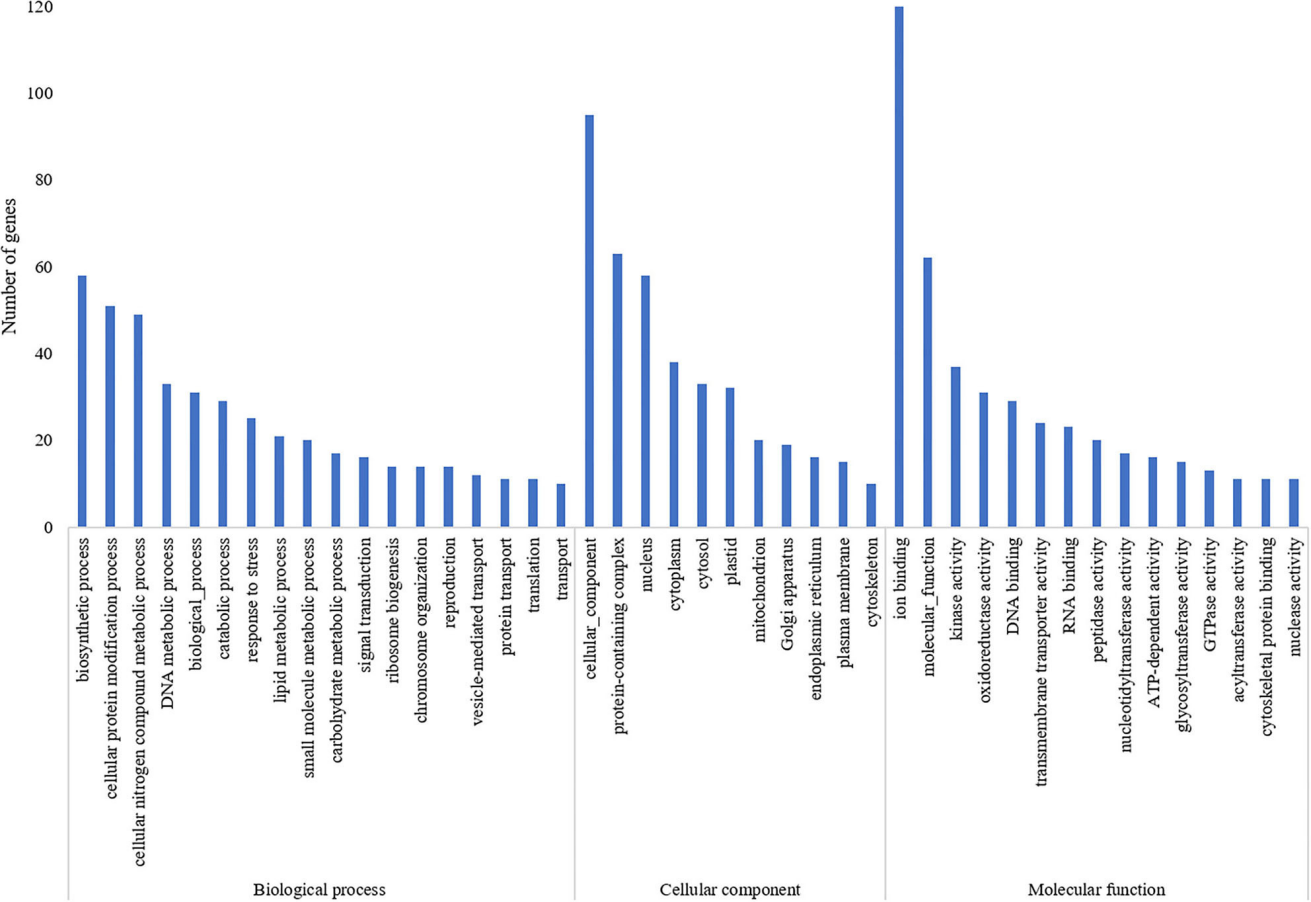
GO terms of genes associated with polymorphic SSRs in black pepper (each term with ≥10 such genes).

**Figure 8:**
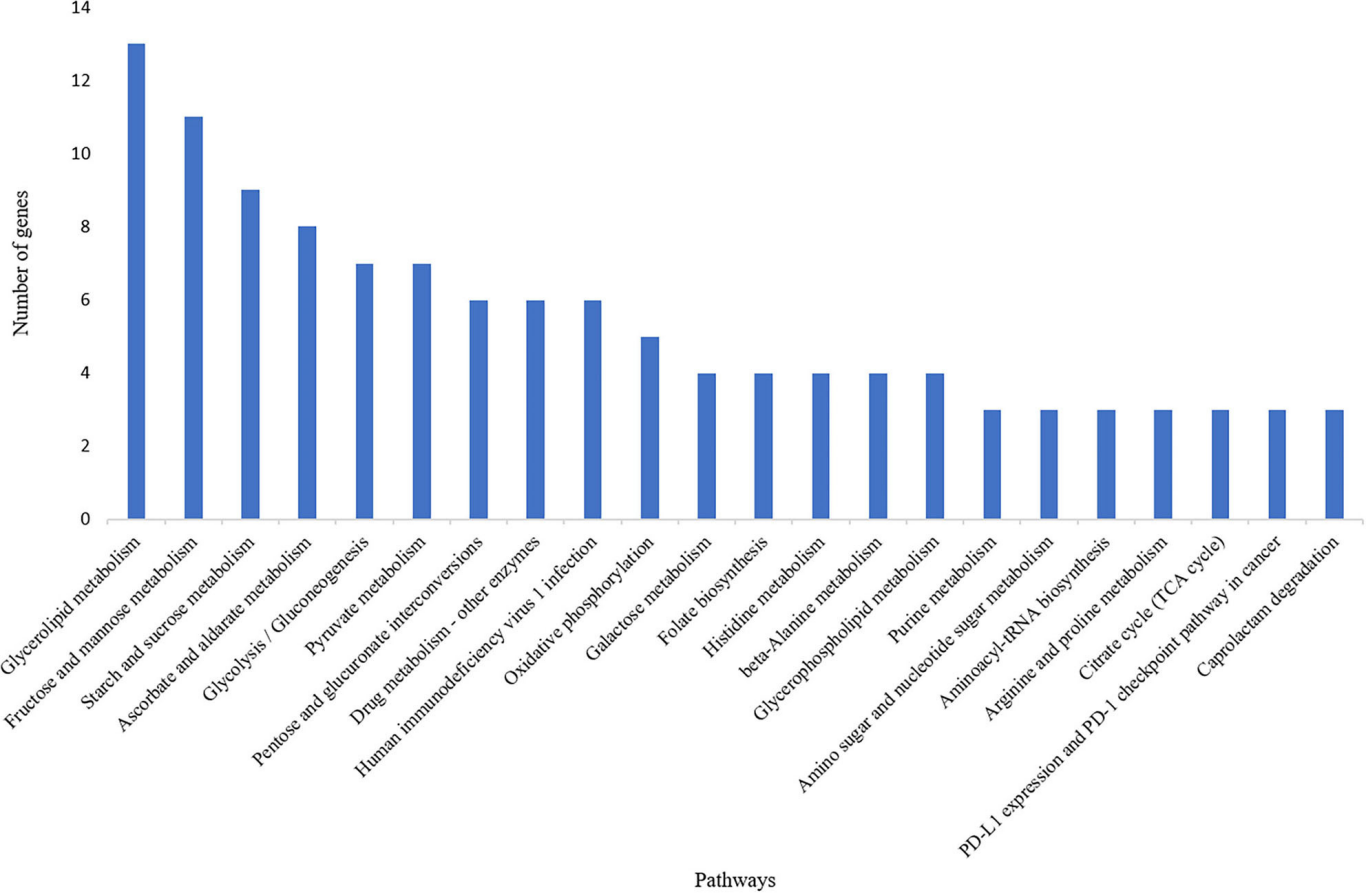
KEGG pathways associated with polymorphic SSRs in black pepper (each pathway with ≥3 such genes).

A total of 835 genes were found associated with polymorphic SSR markers extracted from annotation file of black pepper reference genome. The functional annotation of these gene was performed using Blast2GO and KEGG pathway analyses. A total of 30, 36, and 55 GO terms were found in cellular component, molecular function, and biological process classes, respectively. Figure 7 shows GO terms which were associated with ≥10 genes in all three classes. A total of 67 KEGG pathways were found associated with polymorphic SSRs, out of which pathways associated with ≥3 genes are shown in Figure 8.

### Development of BlackP2MSATdb Database

**Figure 9:**
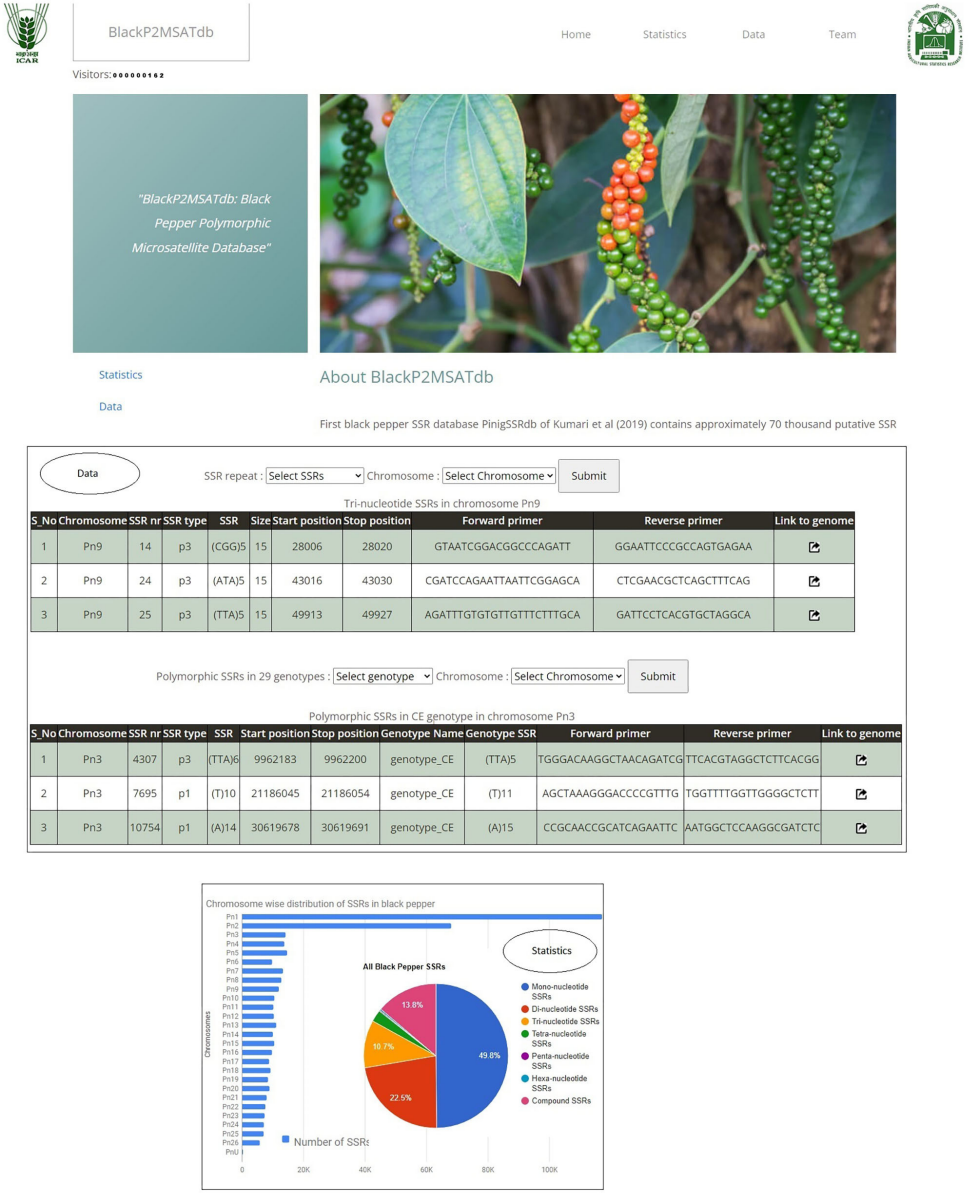
Screenshot of interfaces of BlackP2MSATdb genomic resource.

The Black Pepper Polymorphic Microsatellite Database, *BlackP2MSATdb* genomic resource, is successfully developed using three-tier architecture containing 276,320 SSR loci which are mined from whole-genome sequence data black pepper (Hu et al., 2015) and the GBS data of the 29 black pepper genotypes. It also catalogs 3,176 polymorphic markers which were found polymorphic in 29 genotypes. It has four tabs, *viz.*, Home, Statistics, Data, and Team. The “Home” page has the brief introduction about database. The “Statistics” page included two graphical representation of the proportion of all included data as a sum of all SSRs along with all 3,176 polymorphic SSRs in 29 different black pepper genotypes. The “Data” page navigates the users to get the chromosome-wise SSRs and the motif types. It also provides the polymorphic markers of the 29 black pepper genotypes with respect to the black pepper genome. The “Team” page included the team member name and link to the profile page of each member. The various interfaces of BlackP2MSATdb genomic resource are shown in Figure 9. BlackP2MSATdb is freely available at http://webtom.cabgrid.res.in/blackp2msatdb/.

## Discussion

This is the first comprehensive study for genome-wide microsatellite mining from black pepper latest reference genome recently sequenced (Hu et al., 2019). ddRAD data of 29 black pepper genotypes including released varieties, important germplasm accessions, and wild relatives have been successfully used for discovery of polymorphic markers by computational approach. Similar approach of efficient SSR mining has been reported in other plants also (Xiao et al., 2016). This study is the first in this species, utilizing genomic SSRs for rapid extraction of polymorphic SSRs in other 29 black pepper genotypes to study genetic diversity. Black pepper has immense economic importance world-wide, yet no study till date provides physical localization of black pepper SSRs on 26 chromosomes. Present finding in the form of genomic resources can be used in rapid genetic dissection of complex traits including identification of QTLs for various desired traits, resistance to biotic and abiotic stress, genetic enhancement, and varietal development (Guzmán et al., 2020; Nalbandyan et al., 2020; Younis et al., 2020).

The previous study involving whole-genome SSR mining gave 69126 putative SSR markers utilizing the draft genome comprising of 916 scaffolds and the genome coverage of 80X (Kumari et al., 2019). The total mined SSR markers in the present study are much larger (~4-fold) in number as compared to the previously reported black pepper SSR markers by Kumari et al. (2019), which is due to more than 2-fold depth of the genome assembly being utilized. Around 99.87% of identified SSR markers were localized on 26 chromosomes. Such chromosome and location-specific SSR loci can be further used to trace the inheritance of particular chromosomal regions in molecular breeding programs from the foundation genotypes (Saghai-Maroof et al., 1984; Ganie et al., 2016). SSR markers selected uniformly by taking the advantage of their known physical location over chromosome can be a better diversity calculus for genomic and population variability (You et al., 2005) with respect to random anonymous SSR loci as provided in previous studies (Kumari et al., 2019).

Genomic frequency of identified SSR per Mb and distance between two SSRs suggests that the SSRs are distributed ubiquitously over the entire chromosomes (~362 SSRs/Mb), which can prove to be a better representative for the analysis of genome variability as suggested by Guo et al. (2009). It has the advantage being specific to the location over the targeted chromosome and hence can be used as the marker of any specific genes. Similar density of 341–671 SSRs/Mb has been reported in case of sugar beet (Iquebal et al., 2015).

Identified genomic SSRs with mononucleotide repeats were the most abundant in proportion, followed by dinucleotide and compound repeats. The abundance of mono-repeat was observed in all the genotypes which might be due to the inherent limitation of the next-generation sequencing (NGS) chemistry used for the data generation as reported by Haseneyer et al. (2011). The next abundant dinucleotide motif type has also been widely reported in other crops like wheat, watermelon, *P. dactylifera, E. oleifera*, and *E. guineensis* (Kariin and Burge, 1995; Shioiri and Takahata, 2001; Kumpatla and Mukhopadhyay, 2005; Deng et al., 2016; Zhu et al., 2016).

In the next part of this study, genetic diversity assessment based on black pepper genomic SSRs was performed in 29 black pepper genotypes by rapid extraction of polymorphic SSRs. A total of 3160 genomic SSRs were found to be polymorphic in these 29 genotypes, most of which were intergenic along with promoter and TTS. It is in concordance with the fact that intergenic SSRs have higher utility in DNA fingerprinting and varietal identification in comparison with genic SSRs (Kalia et al., 2011). Similarly, EST-SSRs extracted from expression data were useful for genetic analysis and were found to be relatively low polymorphic and concentrated in genic regions reported in other studies of black pepper (Gordo et al., 2012; Hu et al., 2015). Thus, the present study further strengthens the fact of abundant occurrence of polymorphic SSRs in intergenic region. Functional annotation of genes associated with polymorphic SSRs suggests that the most of these genes were involved in molecular function of various metabolic pathways, as reported in *Curcuma alismatifolia* (Taheri et al., 2019).

The provided method of rapid extraction of polymorphic SSRs could be helpful to extract putative polymorphic SSRs in large group of genotypes. In order to overcome the challenge of traditional way of differentiating varieties with identical morphological characteristics based on phenotypic observation, the revolutionary tool of SSRs has proved to be very effective. They play an important role in identification and development of variety, improvement of trait of interest, QTL mapping, and other diversity analysis. They also aid in the product traceability, the evolutionary, and genetic studies including taxonomic comparison (Zietkiewicz et al., 1994; Iquebal et al., 2013; Yu et al., 2017). The phylogeny of nine black peppers varieties is reported to be determined using the morphological and molecular markers which can be important for breeding scheme (Meilawati et al., 2020).

The catalog of putative SSR markers in BlackP2MSATdb can be a very useful web resource for the varietal differentiation since it also houses the ready-to-use primers required for the rapid genotyping. Literature mentions such approach for varietal differentiation as in case of variety of crops like sugarcane, sesame, capsicum, tea, barley, and eggplant (Karakousis et al., 2003; Manigbas and Villegas, 2004; Stàgel et al., 2008; Sahu et al., 2012; Shirasawa et al., 2013; Bhattacharyya et al., 2014). The web-genomic resource, BlackP2MSATdb, of these extracted genomic and polymorphic SSR markers was prepared to provide access to scientific community to utilize them for further studies involving molecular breeding along with construction of physical map, map-based gene cloning, marker-assisted selection, and evolutionary studies in black pepper and its related species. Also, the developed BlackP2MSATdb resource can be useful regarding the phenomena like “microsatellite mediated enhancement” (MME) of gene expression (Kumar and Bhatia, 2016).

Such chromosome and location-specific SSR loci can be further used to trace the inheritance of particular chromosomal regions in molecular breeding programs from the foundation genotypes (Ganie et al., 2016). The ddRAD method is advantageous in the sense that it is very low-cost and most efficient technique to identify polymorphic SSR markers (Razak et al., 2021). The major limitation of ddRAD, that is, non-uniform coverage of the entire genome, has been successfully overcome in the present work by mapping the SSRs on latest available reference sequence of black pepper (Aballay et al., 2021). The present genomic resource, BlackP2MSATdb having chromosome-wise, location-specific SSR markers, can be of immense use in making of coreset and mapping of genes/QTLs for germplasm management and improvement. The obtained >3 thousand polymorphic SSR markers in this study are very valuable as they are distributed over all the chromosomes. Such markers are imperatively needed for efficient and accurate diversity study and development of varietal signature (Korir et al., 2013). From this work, user can now select equal number of loci from each chromosome for diversity analysis.

We found high degree of polymorphism in all the 29 genotypes with respect to the number of SSRs discovered in each genotype. The obtained >3K polymorphic SSR markers in this study are very valuable as they are distributed over all the chromosomes. Such markers are imperatively needed for efficient and accurate diversity study and development of varietal signature (Bohra et al., 2017). This work clearly demonstrates that ddRAD data are still advantageous in terms of not only rapidity and computational ease but also per unit cost of polymorphic SSR marker discovery which is economical than the whole-genome sequencing. Present finding in the form of genomic resources can be used in rapid genetic dissection of complex traits including identification of QTLs for various desired traits, resistance to biotic and abiotic stress, genetic enhancement, and varietal development (Guzmán et al., 2020; Nalbandyan et al., 2020; Younis et al., 2020).

It was also found in this study that a good number of polymorphic SSRs were hypervariable in reference genome and/or in black pepper genotypes, which is in corroboration of the fact that the hypervariable microsatellites provide a general source of polymorphic DNA markers (Powell et al., 1995). These hypervariable polymorphic SSRs could be utilized in various studies involving diversity analysis, hybrid purity testing, and trait mapping (Marker trait association-MTA) and have been utilized in various previous studies (Singh et al., 2012; Varshney et al., 2012; Dutta et al., 2013; Bohra et al., 2017) in case of pigeon pea.

## Conclusion

The ddRAD-based GBS approach was used for the rapid discovery of genome-wide location-specific polymorphic SSR markers in black pepper. These polymorphic markers were obtained from 29 diverse black pepper genotypes covering the released varieties, important germplasm accessions, and wild relatives. The first comprehensive web-genomic resource of black pepper, BlackP2MSATdb, was developed that catalogs genome-wide deep mining of SSR markers from the genome assembly of black pepper with 26 chromosomes at a single platform. These markers can be searched chromosome-wise along with their physical location. The validation of these markers was done by e-PCR technique by considering all the anonymous wet-lab validated SSRs from published literature. BlackP2MSATdb catalogs a total of 276,230 putative SSR markers identified from the whole genome of black pepper, with relative density 362.88 SSRs per Mb and average distance between two SSRs being 2.76 Kb. A total of 3,176 polymorphic markers were common to black pepper reference genome and 29 genotypes collectively, out of which 2015 were hypervariable. This web-genomic resource can be of immense use in molecular breeding, construction of physical map, identification of QTL, map-based gene cloning, marker-assisted selection, and evolutionary studies in black pepper and its related species.

## Data Availability Statement

The original contributions presented in the study are publicly available. This data can be found at: National Center for Biotechnology Information (NCBI) BioProject database under accession number PRJNA489154.

## Author Contributions

SJ, MI, JK, and DK conceived theme of the study and designed experiment. JK and PU collected the samples. KS, AN, MI, SJ, and UA were involved in computational analysis and development of web resources. AR collected resources. SJ, MI, DK, and AR supervised the study. AN, KS, SJ, and MI were involved in writing the original draft. DK and AR reviewed and edited the manuscript. All authors read and approved the final manuscript and contributed to the article and approved the submitted version.

## Conflict of Interest

The authors declare that the research was conducted in the absence of any commercial or financial relationships that could be construed as a potential conflict of interest.

## Publisher's Note

All claims expressed in this article are solely those of the authors and do not necessarily represent those of their affiliated organizations, or those of the publisher, the editors and the reviewers. Any product that may be evaluated in this article, or claim that may be made by its manufacturer, is not guaranteed or endorsed by the publisher.

## Supplementary Material

Supplementary File 1 Black pepper variety details.

Supplementary Table S1 SSR motifs and their frequency for di- (including all motifs), tri- (including motifs with frequency ≥141), tetra- (including motifs with frequency ≥10), penta- (including motifs with frequency ≥3), and hexa-nucleotide (including motifs with frequency ≥2) type.

Supplementary Table S2 Set of validated microsatellites used for *in silico* re-validation.

## Abbreviations

SSR: simple sequence repeats
QTL: quantitative trait loci
DNA: deoxyribonucleic acid
GBS: genotyping-by-sequencing
CTAB: cetyltrimethylammonium bromide
HS: high sensitivity
MISA: MIcroSAtellite identification
GC: guanine–cytosine
TTS: transcription termination sites
HOMER: Hypergeometric Optimization of Motif EnRichment
BLAST: Basic Local Alignment Search Tool
KEGG: Kyoto Encyclopedia of Genes and Genomes
NGS: next-generation sequencing.
