# Supplementary material for: Rapid Genome-Wide Location-Specific Polymorphic SSR Marker Discovery in Black Pepper by GBS Approach
Source: Front Plant Sci. 2022 May 27;13:846937. doi: 10.3389/fpls.2022.846937 (PMC9197322; doi:10.3389/fpls.2022.846937)
Supplement: Supplementary Table S1 — SSR motifs and their frequency for di- (including all motifs), tri- (including motifs with frequency ≥141), tetra- (including motifs with frequency ≥10), penta- (including motifs with frequency ≥3), and hexa-nucleotide (including motifs with frequency ≥2) type. [file Table_2.DOCX]

**Supplementary Table S1**. SSR motifs and their frequency for di (including all motifs), tri (including motifs with frequency ≥141), tetra (including motifs with frequency ≥10), penta (including motifs with frequency ≥3) and hexa-nucleotide (including motifs with frequency ≥2) type.

| Di | | Tri | | Tetra | | Penta | | Hexa | |
| --- | --- | --- | --- | --- | --- | --- | --- | --- | --- |
| Motif | Freq.* | Motif | Freq. | Motif | Freq. | Motif | Freq. | Motif | Freq. |
| TA | 27311 | AAT | 4219 | AATA | 1337 | AAAAT | 81 | CCGATT | 323 |
| AT | 24802 | TTA | 4132 | TATT | 1165 | TTTAT | 48 | GAATCG | 253 |
| TG | 1750 | ATT | 1843 | TTTA | 1109 | TATTT | 39 | TCGGAA | 59 |
| AG | 1632 | ATA | 1826 | ATAA | 864 | ATTTT | 27 | GGAATC | 33 |
| AC | 1567 | TAA | 1660 | AAAT | 749 | CGAGC | 24 | AATCGG | 27 |
| CT | 1223 | TAT | 1518 | TTAT | 596 | TAAAA | 21 | GATTCC | 20 |
| TC | 1141 | AAG | 1422 | ATTT | 246 | ATAAA | 21 | CGATTC | 11 |
| CA | 1011 | TCT | 1380 | TAAA | 225 | TTTTA | 20 | AAAATA | 8 |
| GT | 800 | GAA | 1101 | ATTA | 102 | AAATA | 17 | AAAAAT | 5 |
| GA | 716 | TTC | 877 | TAAT | 95 | AATAA | 17 | ATTCCG | 5 |
| GC | 95 | CGC | 785 | TTAA | 80 | TTATT | 17 | CTCCCG | 4 |
| CG | 34 | GGC | 632 | AATT | 52 | CCCGC | 14 | TCCTAT | 4 |
| - | - | CCG | 626 | AAAG | 50 | GCTCG | 14 | GCGGGA | 4 |
| - | - | CTT | 597 | ATGT | 28 | TCGGC | 10 | TATTTT | 3 |
| - | - | CGG | 593 | ACAT | 27 | GCCCC | 9 | TTTATT | 3 |
| - | - | GCG | 542 | TATG | 27 | GGCGG | 9 | AAATAA | 3 |
| - | - | AGA | 490 | TTTC | 27 | ATCTC | 7 | GGTGGC | 3 |
| - | - | CAA | 446 | CCGC | 19 | TATTA | 7 | CGGAAT | 3 |
| - | - | GCC | 391 | TTCT | 18 | GGGGC | 7 | CTCAAT | 3 |
| - | - | TTG | 306 | ATAG | 18 | GAGGG | 6 | CTCTCC | 2 |
| - | - | TGT | 296 | CATA | 16 | GGGCG | 6 | AGAAGG | 2 |
| - | - | GGA | 224 | AACA | 15 | ATAAT | 6 | GGGGAA | 2 |
| - | - | GAG | 193 | AAGA | 15 | CGCCC | 5 | GACGGC | 2 |
| - | - | GGT | 191 | ATAC | 15 | ATATA | 5 | GGGGAG | 2 |
| - | - | CCT | 178 | GGAG | 15 | ATTAT | 4 | GAGATG | 2 |
| - | - | CCA | 173 | TCTT | 14 | TTATA | 4 | CAACTC | 2 |
| - | - | TCC | 170 | TTTG | 14 | TATAA | 4 | TTTTTA | 2 |
| - | - | CTC | 162 | GTAT | 13 | GAAAT | 3 | GAGAGG | 2 |
| - | - | GAT | 160 | AGAA | 12 | TTAAT | 3 | TCTGTG | 2 |
| - | - | AAC | 152 | GGGC | 11 | GAGCC | 3 | TGGGAA | 2 |
| - | - | ATG | 148 | AAAC | 10 | GAGAT | 3 | AGACAC | 2 |
| - | - | ATC | 141 | GGCG | 10 | CCTCT | 3 | CGGCGA | 2 |
| - | - | AGG | 141 | TATC | 10 | CCCCA | 3 | CTATAT | 2 |

*Freq.=Frequency
