# Supplementary material for: Rapid Genome-Wide Location-Specific Polymorphic SSR Marker Discovery in Black Pepper by GBS Approach
Source: Front Plant Sci. 2022 May 27;13:846937. doi: 10.3389/fpls.2022.846937 (PMC9197322; doi:10.3389/fpls.2022.846937)
Supplement: Supplementary Table S2 — Set of validated microsatellites used for in silico re-validation. [file Table_3.DOCX]

Supplementary Table S2: Set of validated microsatellites used for in silico re-validation

| **Reference** | ***In silico* validated SSR primers** |
| --- | --- |
| Wu et al., 2016 | PE06:CTT(5), PN01:GT(9), PN03:CAT(7), PN04:CTC(7) |
| Kumari et al., 2019 | Pn12:AT, Pn21:TAT, Pn35:TTA, Pn41:(TA)T(TA), Pn49:(AT)A(AT), Pn50:(TC)(TA), Pn51:AT, Pn55:(TTC)(TCT), Pn56:(TA)(TTTA), Pn61:(TC)(AC), Pn64:TA, Pn67:TA, Pn70:GCG, Pn71:AAT, Pn72:TTA, Pn75:TTA, Pn78:ATA, Pn80:(TG)(TA), Pn84:CCT |
| Menezes et al., 2009; | PNA5:(AC)19, PNB5:(TG)14, PNE3:(CA)13, PNG11:(AC)5 |
| Raghavan et al., 2010; | PNA5:(AC)19, PNB5:(TG)14, PNE3:(CA)13, PNG11:(AC)5 |
| Jagtap et al., 2016 | PNA5:(AC)19, PNB5:(TG)14, PNE3:(CA)13, PNG11:(AC)5 |
| Joy et al., 2011 | PnAG30:(CT)4TT(CT)16, PnGT119:(AT)6(GT)24 |
| Jose et al., 2017 | PNS8:(TCT)5 |
| Hu et al., 2015 | CL10038,CL10084,CL10176,CL10451,CL10564,CL10615,CL10651,CL10717,CL10739,CL10785,CL1080,CL10821,CL10878,CL10969,CL11100,CL11122,CL1117,CL11225,CL11229,CL11272,CL11573,CL11620,CL11639,CL11644,CL11723,CL11845,CL11894,CL11897,CL11906,CL11921,CL1203,CL12034,CL12129,CL12191,CL12300,CL12349,CL1240,CL12478,CL12490,CL1257,CL1261,CL12764,CL1289,CL12903,CL13006,CL13097,CL1316,CL13162,CL132,CL13369,CL13419,CL13438,CL13634,CL13734,CL13766,CL13838,CL13843,CL13954,CL13997,CL14055,CL1409,CL14177,CL14236,CL14340,CL14426,CL1450,CL14513,CL14524,CL14894,CL14966,CL15071,CL15144,CL15231,CL15279,CL15678,CL15772,CL15837,CL16011,CL16121,CL16272,CL16365,CL16400,CL16411,CL16416,CL16637,CL16693,CL16740,CL1680,CL16869,CL16896,CL16976,CL17100,CL17159,CL17162,CL1818,CL196,CL2059,CL2072,CL2087,CL2140,CL2272,CL2315,CL2364,CL242,CL2522,CL2585,CL2604,CL2685,CL2733,CL2883,CL3026,CL3080,CL3246,CL3271,CL3275,CL3310,CL3320,CL3481,CL3624,CL3764,CL3795,CL3995,CL4031,CL4066,CL4077,CL4084,CL4103,CL4139,CL4166,CL423,CL4459,CL4460,CL4522,CL4562,CL4655,CL4803,CL4805,CL5050,CL5080,CL5115,CL5179,CL5290,CL5363,CL5696,CL5697,CL57,CL5782,CL5820,CL6072,CL6443,CL6532,CL6534,CL6682,CL6687,CL6708,CL6952,CL697,CL7090,CL7099,CL7195,CL7225,CL7232,CL728,CL7313,CL7335,CL7359,CL7447,CL7518,CL7523,CL7698,CL7724,CL7746,CL7814,CL785,CL7859,CL7961,CL7983,CL8044,CL8105,CL8108,CL8112,CL8137,CL816,CL8229,CL8248,CL8250,CL8342,CL8409,CL8533,CL8570,CL8657,CL8779,CL8936,CL8938,CL8988,CL9209,CL9263,CL9391,CL9418,CL9442,CL9507,CL9584,CL9637,CL9663,CL9676,CL9908,CL9912,CL9921,CL996,Unigene10008,Unigene1006,Unigene10390,Unigene10604,Unigene10813,Unigene10878,Unigene10889,Unigene10893,Unigene10905,Unigene11063,Unigene11326,Unigene1138,Unigene11418,Unigene11507,Unigene11668,Unigene11688,Unigene11800,Unigene11827,Unigene11899,Unigene11938,Unigene1197,Unigene11980,Unigene11985,Unigene121,Unigene12208,Unigene12488,Unigene12562,Unigene12817,Unigene1283,Unigene13185,Unigene13264,Unigene13274,Unigene13363,Unigene13514,Unigene1361,Unigene13745,Unigene13787,Unigene13896,Unigene14392,Unigene1447,Unigene14489,Unigene1464,Unigene14694,Unigene1475,Unigene14759,Unigene14801,Unigene14888,Unigene15033,Unigene15064,Unigene15117,Unigene15241,Unigene15620,Unigene16022,Unigene16036,Unigene16197,Unigene16293,Unigene1646,Unigene16708,Unigene16981,Unigene17318,Unigene17506,Unigene17508,Unigene1777,Unigene17910,Unigene18696,Unigene18787,Unigene19068,Unigene19099,Unigene19425,Unigene20542,Unigene20705,Unigene2173,Unigene22634,Unigene24624,Unigene25027,Unigene26438,Unigene287,Unigene30907,Unigene333,Unigene350,Unigene370,Unigene37476,Unigene43784,Unigene4410,Unigene4582,Unigene5287,Unigene535,Unigene5359,Unigene5623,Unigene5769,Unigene5793,Unigene5852,Unigene5916,Unigene6114,Unigene6183,Unigene629,Unigene6721,Unigene676,Unigene7071,Unigene7116,Unigene7123,Unigene7288,Unigene756,Unigene7684,Unigene77,Unigene8181,Unigene823,Unigene8507,Unigene8783,Unigene8792,Unigene8832,Unigene8860,Unigene8904,Unigene8980,Unigene9157,Unigene9215,Unigene9535,Unigene9740,Unigene9792,Unigene9884,Unigene9918 |
